# The impact of different types of parental support behaviours on child physical activity, healthy eating, and screen time: a cross-sectional study

**DOI:** 10.1186/s12889-016-3245-0

**Published:** 2016-08-24

**Authors:** Evelyn Pyper, Daniel Harrington, Heather Manson

**Affiliations:** 1 Public Health Ontario, 480 University Avenue, Suite 300, Toronto, Ontario M5G 1V2 Canada; 2 School of Public Health and Health Systems, University of Waterloo, 200 University Avenue West, Waterloo, Ontario Toronto, N2L 3G1 Canada; 3 Dalla Lana School of Public Health, University of Toronto, 155 College Street, 6th floor, Toronto, Ontario M5T 3M7 Canada

**Keywords:** Parent support behaviours, Child obesity, Physical activity, Healthy eating, Screen time

## Abstract

**Background:**

In Canada, 31.5 % of children are overweight or obese, putting them at an increased risk of chronic co-morbidities and premature mortality. Physical activity, healthy eating, and screen time are important behavioural determinants of childhood overweight and obesity that are influenced by the family environment, and particularly parents’ support behaviours. However, there is currently a limited understanding of which types of these support behaviours have the greatest positive impact on healthy child behaviours. This study aims to determine the relative contribution of different types of parental support behaviours for predicting the likelihood that children meet established guidelines for daily physical activity, daily fruit and vegetable consumption, and recreational screen time.

**Methods:**

A Computer Assisted Telephone Interview survey was used to collect data from a random sample of parents or guardians with at least one child under the age of 18 in Ontario (*n* = 3,206). Three multivariable logistic regression models were built to predict whether or not parents reported their child was meeting guidelines. Independent variables included parent and child age and gender, multiple indicators of parental support behaviours, and socio-demographic characteristics. Parental support behaviours were categorized post-hoc as motivational, instrumental, regulatory, and conditional based on an adapted framework.

**Results:**

Controlling for all other factors in the model, several parental support behaviours were found to be significant predictors of children meeting established health guidelines. For example, conditional support behaviours including taking the child to places where they can be active (OR: 2.06; 95 % CI: 1.32-3.21), and eating meals as a family away from the TV (95 % CI: 1.15-2.41) were significant positive predictors of children meeting physical activity and fruit and vegetable guidelines, respectively.

**Conclusions:**

Health promotion efforts aimed at improving particular parent support behaviours could be effective levers for mitigating the burden of excess body weight in childhood. As such, the influence of support behaviours should be fully considered in any comprehensive approach to prevention and reduction of childhood overweight and obesity.

## Background

Addressing obesity and overweight in children and youth (under age 18) is a key public health priority in many industrialized countries. In Canada, over a period of twenty-five years (1978–79 to 2004), rates of obesity in children more than doubled, while rates of overweight increased by approximately 29 % [1, 2]. The most recent estimates derived from direct anthropometric measurements (i.e., Body Mass Index (BMI) calculated from weight and height) indicate that 31.5 % of Canadian children and youth are now overweight or obese according to World Health Organization (WHO) cut-offs [3]. This estimate is similar to those in other countries in the western world [4, 5]. These rates indicate a significant public health issue given that overweight and obese individuals are at an increased risk of chronic conditions in adulthood (e.g., cardiovascular disease, type II diabetes, cancer), and ultimately an increased risk premature mortality [6].

At the physiological level, the accumulation of fat on the body is caused by an energy imbalance whereby caloric intake from diet exceeds the calories expended through human metabolism and physical movement. As such, physical activity, sedentary behaviour, and healthy eating are important behavioural determinants of overweight and obesity [7–9]. Given their importance, each of these behaviours has its own set of national guidelines in Canada, each of which has been developed to reflect the changing needs of children as they age (Table 1). The relationships between these behaviours and body weight cannot be assumed to be linear, and rather, result from complex interactions between individuals and the physical, social, political and economic environments in which they live (e.g., school, workplace, home, neighbourhood) [10]. For children and youth, mediating variables in the relationship between health behaviours and body weight extend to the influence of parents through means such as parental modelling, parental support behaviours, and the family environment [11]. A successful response to child and youth overweight and obesity must consider these underlying complexities [12].

**Table 1:** Guidelines for Child Health Behaviours

Behaviour	Source	Child’s Age	Guideline/Recommendation
Physical Activity	*Canadian Physical Activity Guidelines* from the Canadian Society for Exercise Physiology (CSEP)	<1 years	Be physically active several times daily
		1-4 years	Accumulate at least 180 min of physical activity at any intensity throughout the day
		5-17 years	Accumulate at least 60 min of moderate- to vigorous-intensity physical activity daily
Healthy Eating	*Canada’s Food Guide* from Health Canada	2-3 years	4 fruit & vegetable (FV) servings per day
		4-8 years	5 FV servings per day
		9-13 years	6 FV servings per day
		14-18 years	*Females*: 7 FV servings per day
			*Males*: 8 FV servings per day
Screen Time	*Canadian Sedentary Behaviour Guidelines* from the Canadian Society for Exercise Physiology (CSEP)	<2 years	Screen time is not recommended
		2-4 years	Screen time should be limited to under 1 h per day
		5-17 years	Limit recreational screen time to no more than 2 h per day

### Parental support for child health behaviours

Children’s health behaviours are highly influenced by their parents, with whom children are in close proximity for the early part of their lives [13]. For example, parental support behaviours have been found to correlate closely with the physical activity, eating behaviour, and body weight of preschool and school-aged children [14, 15]. Behaviours of children under 12 years of age are under less volitional control than older children, and thus, parents play a major role in promoting or inhibiting opportunities for healthy active living [16]. Although adolescents may increasingly assert autonomy over their behaviours [17], a strong relationship remains between parental social support and adolescent physical activity [18]. These findings align with results indicating that children’s health behaviours are shaped by their parents throughout their formative years, and even as they enter and proceed through adolescence [18–24].

Parents have been described as *gate-keepers* of children’s health-related behaviours, through their provision of social support for physical activity [25] and healthy eating [26]. In acknowledgement of the considerable variation in how social support is operationalized in the literature, Beets and colleagues [16] propose the following physical activity-specific definition:

“Social support represents the functional characteristics associated with the interactions between a parent and his or her children in the context of intentionally participating in, prompting, discussing, and/or providing activity-related opportunities.” (p. 624).

This definition of social support as an umbrella term is used as a frame for the purposes of this paper as it can be applied to parental support for three specific child health behaviours: physical activity, healthy eating, and screen time.

### Physical activity

Physical activity may be promoted or inhibited by the behaviours of parents and the family environment [27]. There is evidence to suggest that targeting parental support behaviours directly, as opposed to other constructs (e.g., attitudes) can be effective strategies for improving children’s physical activity [28]. That is, many parents are already convinced of the importance of physical activity for their children, and when attitudes towards health behaviours are ceilinged, there is little room for improvement [29].

In order to target parental support behaviours effectively, the relative contributions of specific parental support behaviours to child health must be better understood. There are a number of parent behaviours that have been found to be associated with increasing child physical activity [20, 21, 30]. Parents’ own physical activity levels as observed by their children—often referred to as parental modelling—have been shown to be positively associated with children’s physical activity levels [19, 30–32]. Others have proposed that parent activity is insufficient to increase children’s physical activity. Trost and colleagues [33] as well as others [34] have suggested that children’s physical activity may instead be more strongly determined by parents’ support behaviours across a variety of strategies. Positive reinforcement (e.g., encouragement) has been found to be significantly related to child and adolescent physical activity, perhaps by increasing children’s feelings of competency and behavioural intentions [35]. Providing transportation and equipment, as well as enrolling children in opportunities for physical activity, represent instrumental parental support behaviours associated with increased physical activity in children [36]. Finally, evaluations of interventions have shown that parent involvement in children’s physical activities (e.g., shared participation) improves intervention success; yet, evidence based on interventions exclusively targeting families are limited and inconclusive [37].

### Healthy eating

Children’s eating patterns are also influenced by a variety of family and social factors, with parents’ behaviours playing a direct role [38]. Eating patterns may refer to various mealtime characteristics (e.g. where the meal is eaten and who the meal is eaten with) or indicators of diet quality including meeting food-based guidelines or recommendations [38]. Independent of the healthy eating metric (e.g., fruit and vegetable (FV) consumption, dietary fat intake, consumption of sugar-sweetened beverages), it is important to consider the influence of a range of parental support behaviours. Lopez et al. [39] demonstrated that greater parental support for healthy eating in children, aged 5 to 8 years old, is associated with less consumption of sugar-sweetened beverages, where parental support was represented by a composite score based on: (1) encouraging the child to eat FV; (2) providing fruit or vegetables to the child; (3) eating FV with the child; (4) encouraging the child not to drink sugary beverages; and, (5) talking with the child about correct food portion sizes. Despite these findings, the relative influence of specific types of parental support behaviours cannot be elucidated when variables are collapsed into one summary score (e.g., a composite index). When parental support behaviours are examined separately, parental encouragement to eat healthy foods has been shown to have a significant positive influence children’s dietary behaviours [15, 40].

Instrumental support (e.g., purchasing a child a new fruit to try) has been associated with FV availability and accessibility, which can influence children’s eating environment, and thereby shape children’s eating patterns [26]. Where children eat their meals is also relevant, as eating out at restaurants is associated with higher intake of dietary fat and energy compared to eating at home [38]. Eating out at fast-food restaurants at least weekly is also associated with greater consumption of sugar-sweetened beverages in children aged 5 to 8 years old [39]. Moreover, children’s eating patterns can be influenced by the social context of meals, specifically whether their family eats together. Children who have dinner together with family members tend to eat more healthy foods and nutrients [38]. Similarly, children who share family meals three or more times per week are more likely to be in a normal weight range and have healthier dietary patterns than those who share fewer than three family meals together [41]. Alternatively, when TV-viewing is a normal part of the family eating experience, children tend to consume more pizza, snack foods, and sodas, and fewer FV than those who do not watch TV during meals [39, 42].

### Screen time

The past decade has seen an influx of screen-based sedentary pursuits above and beyond TV-watching, including cell phones, tablets, and video games [43]. There is now a greater understanding of the harmful nature of sedentary behaviour, and consequently, current recommendations call for individuals to not only *move more*, but also *sit less* [43]. TV viewing and other types of screen time act as proxy measures for sedentary behaviour, which is characterized by little physical movement and low energy expenditure [8, 44]. Saunders and colleagues [45] found that short bouts of sedentary behaviour (e.g., those lasting 1–4 minutes) and breaks in sedentary time are associated with reduced cardiometabolic risk in children, aged 8 to 11 years old, independent of total sedentary time and physical activity. Further, they suggest that TV viewing and leisure time computer and video gaming presented risks independent of objectively measured sedentary time [45]. In other words, time spent sitting is bad for health, but time spent sitting in front of a screen is worse. While both peer and family relationships influence children’s health behaviours, previous work suggests that family relationships may be more important for decreasing screen-based sedentary behaviours such as TV/video viewing and computer/video game playing in 10- to 14-year-old girls [22].

Consideration of specific parental factors reveals that parent TV viewing is associated with child TV viewing across all ages when controlling for media access variables, parent co-viewing, time restrictions, parental characteristics, and demographic/household characteristics. Specifically, every hour of parent TV viewing resulted in an additional 23 min of child TV viewing [46]. In addition to modelling, observational learning is likely to occur via co-viewing, whereby parents watch TV with their children [47]. Socioecological theory proposes that TV screen time may be an integral part of family life [48], and thus, co-viewing may be a key component of a family’s shared time. Nonetheless, parental co-viewing is associated with increased TV time among young children and adolescents [23, 46, 49]. A possible explanation is that co-viewing does not give children another model of how to spend their leisure time, inadvertently ingraining screen time habits [23, 50].

Presence of rules has been associated with a lower probability of excessive spending time on screen-based activities by 11- and 15-year-old children; however, rule-setting to restrict the content of TV programs or computers showed no association [23]. The extent of parents’ screen time rule-setting has been shown to vary depending on the age of the children, parental age, and parental race [51, 52]. Moreover, it is important to consider screen time messages children receive from parents’ verbal communication and how these might differ from parents’ own actions [53].

### Towards an integrated classification of parental support behaviours

Despite often being studied in isolation, it is evident that child physical activity, healthy eating, and screen time are inter-related. For example, screen time has an independent contribution to overweight and obesity, but may also contribute through other behavioural mechanisms by: decreasing time spent engaging in physical activity [54]; increasing snacking while viewing [55]; and interrupting sleep duration [56, 57]. Likewise, each of these can be influenced by parental support behaviours, and there are clearly similarities in the types of supports used by parents across child health behaviours.

A consensus on how to classify parental support behaviours is currently lacking in the healthy eating and screen time literature. Even in the physical activity literature, where operationalizations of social support behaviours have received some focus, there is great variation in how behaviours are classified [16, 58]. In this vein, a limitation of many studies is the combination of parental support behaviours into a composite measure; thus, the specific effects of different parental supports cannot be investigated [16, 59].

In recognition of the inconsistencies in parental support behavior classifications [58], Beets et al. [16] identify categories of social support for youth physical activity-related behaviours. *Motivational*, *instrumental*, and *conditional* parental support are clearly defined by the Beets et al. [16] framework that provides a foundation for the classification scheme used for this study (Table 2). To account for behaviours that involve enforcing rules or setting limits, which we consider a unique parental support behaviour, we have adapted this framework to include a *regulatory* category in the classification scheme.

**Table 2:** Types of Parental Support Behaviours, Adapted from Beets et al. [16]

Type	Motivational	Instrumental	Regulatory	Conditional
Definition	Provision of verbal/nonverbal prompts to engage in the behavior of interest, validation and affirmation of involvement or performance from participating in the behavior.	Provision of tangible aid and/or services	Enforcing rules and/or setting limits	Directly involved in, or within proximity of, the activity with the child

Knowing *what* promotes child health is futile if we do not know *how* to best support these health behaviours. Establishing a classification scheme of four defined types of parental support behaviours allows for inquiries of the impact of these both between and within different types of health behaviours. Related findings would be useful for the development of health promotion activities across a variety of settings, including communications strategies and messages directed at parental support behaviours.

### Objectives

The purpose of this study is to determine the relative contribution of different types of parental support behaviours for predicting whether children are meeting recommendations for physical activity, screen time, and healthy eating. Specific objectives are: (1) to classify types of parental support behaviours using the classification scheme identified in Table 2; (2) to determine if the different types of parental support behaviours predict child health behaviours; and, (3) to assess these relationships within and between different child health behaviours. This study aims to fill the existing gap in knowledge within the literature regarding the relative influence of specific types of parental support behaviours on child health behaviours; and to provide evidence to inform the development of population-level interventions, and other initiatives to address child health behaviours by modifying parental support behaviours.

## Methods

### Data

This study uses data collected from a Computer Assisted Telephone Interview (CATI) survey with parents/guardians in Ontario who felt comfortable answering questions about the child in the household with the next birthday. A random sample of publically available phone numbers was drawn, including both landlines and cellular phones. Data were collected from parents or guardians with at least one child under the age of 18 (*n* = 3,206). Due to the length of the survey, parents were asked to answer at least two of the four health behaviour modules (physical activity, healthy eating, recreational screen time, and sleep). These were randomly selected at the time of the interview. Participants were also invited to complete additional, optional modules at the end of the survey.

Of the total number of respondents, 2,237 answered the physical activity module, 2,288 answered the healthy eating module, 2,248 answered the screen time module, and 2,227 answered the sleep module. All respondents answered questions about community perceptions, and demographics. The survey achieved a response rate of 7 %^1^ and a cooperation rate of 12 %^2^ based on American Association for Public Opinion Research standard definitions [60].

Dependent variables were derived as follows: For physical activity, parents were given a definition of moderate to vigorous physical activity (MVPA), and were asked to report the average minutes of MVPA their child got each day in the past week. For the indicator of healthy eating, parents were asked to report how many FV servings their child eats on a typical day. Children under the age of two were excluded from the analysis, because there are no behavioural data for children under the age of one, and there is no FV guideline for children under two. Finally, parents were asked to how long their child spends, on a typical day, using the following items (not including time spent doing homework, or time spent in front of a screen at school): television and/or DVD player; computer or laptop; tablet or iPad®; and video game console. Total number of screen time minutes were calculated by adding these four items. Children under the age of one were excluded from the analysis, as no behavioural data are available for this group. Sleep was not included in this study because of the lack of established national guidelines related to sleep hygiene. Average daily MVPA, average daily FV servings, and average daily screen time during leisure time were compared to established behavioural guidelines for age and gender (Table 1). This process resulted in the construction of binary dependent variables (i.e., *meets guideline* versus *does not meet guideline*) for each of the three child health behaviours.

Key independent variables for this study are the different types of parental support behaviours related to each child health behaviour. Parents were asked to indicate whether they *strongly agree*, *agree*, *neither agree or disagree*, *disagree*, or *strongly disagree* with each of the statements found in Table 3, which have been organized according to the adapted framework presented in Table 2. To create binary independent variables, the responses *strongly agree* and *agree* were collapsed into a single item, as were the remaining responses. Child age was re-coded from a continuous variable to a categorical variable with the following age groups: 0 to 4 years old, 5 to 8 years old, 9 to 12 years old, and 13 to 17 years old. A binary variable was created for marital status, comprised of *living with partner* (living common-law, married) and *not living with partner* (widowed, separated, divorced, single). Time since immigration was re-coded to become *0 to 9 years* (0–4 years ago, 5–9 years ago), *10+ years* (10–20 years ago, more than 20 years ago), and *Canadian-born*. The variable measuring parents’ highest level of education completed was re-coded as follows: *below secondary school* (never attended school, less than secondary school graduation), *secondary school diploma*, and *post-secondary school* (apprenticeship or trades certificate or diploma; college, CEGEP or other non-university certificate or diploma; university certificate or diploma below bachelor level; bachelor’s degree; university certificate, diploma or degree above bachelor level). Employment status was also re-coded into the following categories: *employed* (full-time, part-time, self-employed), *unemployed*, and *other* (Ontario Disability Support Program, retired, student, homemaker, working without pay, other). Total household income categories were re-coded from increments of $10,000 to increments of $20,000. It should be noted that those who refused to report their income were treated as their own category (*did not respond*). Finally, the variable measuring number of children in the household was re-coded into *1*, *2 to 3*, and *4 or more* categories.

**Table 3:** Parental support behaviour domains, by type of support

Type	Motivational	Instrumental	Regulatory	Conditional
Physical Activity	• I encourage my child to walk or cycle to places (e.g., friend’s houses, school) if they are reasonably close • I encourage my child to use resources in our community to be active (e.g., parks) • I encourage my child to be active outdoors with friends and family	• I enroll my child in sports teams, clubs, or community-based programs where < HE/SHE > can be active (e.g., Boys & Girls club, YMCA) • I take my child to places where < HE/SHE > can be active		• I take part in physical activities with my child • I watch my child play sports or do other activities (e.g., martial arts, dance)
Healthy Eating	• I encourage my child to help choose and prepare snacks and meals • I encourage my child to eat breakfast	• I serve raw vegetables and/or fruit for snacks between meals		• I eat meals prepared or purchased at fast food restaurants with my child • We eat meals as a family away from the TV
Screen Time	• I encourage my child to limit their screen time during leisure time		• I enforce rules about my child’s screen time	• My family watches TV together

### Statistical analysis

All analyses were conducted with SAS Version 9.3. Descriptive statistics were calculated to describe the sample by demographic characteristics, parental support behaviours, and child health behaviours. Bivariate analyses were used to determine the relationships between all independent variables (including the different types of parental support behaviours) and dependent variables (whether children reach the guidelines) for each of the health behaviours. Specifically, independent samples *t*-tests were conducted for continuous variables and chi-square tests were conducted for categorical variables. Independent variables included: child age and gender, parent age, gender, marital status, time since immigration, education, employment status, total household income, and number of children in the household.

To determine the relative importance of parent support behaviours in predicting whether children meet health guidelines, multivariable logistic regressions were used. Models were built starting with parent and child age and gender; followed by the inclusion of parental support behaviours; followed by socio-demographic characteristics (marital status, time since immigration, highest level of education completed, employment status, total household income, and number of children in household). Based on indications of its influence on child health behaviours [19], a parent–child gender interaction term was tested. These were found to be non-significant predictors for all three child health outcomes, and were therefore excluded from the models. Similarly, a variable measuring child physical or mental condition that may limit activities, as well as household-level food insecurity were not significant predictors of meeting physical activity, or FV guidelines, respectively. Thus, these variables were also excluded from final logistic regression models due to lack of significance. Generalized variance inflation factors did not indicate multicollinearity in any of the regression models. Differences between estimates were tested for statistical significance at *p* < 0.05.

## Results

### Sample

Male (*n* = 1620, 50.6 %) and female children (*n* = 1585, 49.5 %) were represented with a relatively even distribution of ages ranging from 0 to 17 (mean = 8.83, sd = 5.23, median = 9). Conversely, parents were predominantly female (71.0 %) with ages ranging from 19 to 73 (mean = 41.92, sd = 8.10, median = 42). Demographic characteristics and other variables describing the study sample can be found in Table 4.

**Table 4:** Sample Characteristics of Study Population

	Sample		Ontario Population^a^	
Variable	N	%	N	%
Number of respondents	3206	100	2693850	100
Child age category (years)				
0 to 4	851	26.6	704260	26.1
5 to 8	686	21.5	569275	21.1
9 to 12	683	21.4	590620	21.9
13 to 17	977	30.6	829695	30.8
Child gender Male	1620	50.6	1381630	51.3
Female	1585	49.5	1312220	48.7
Parent gender				
Male	930	29.0	--	--
Female	2274	71.0	--	--
Marital status				
Not living with partner	469	14.7	604645	16.7
Living with partner	2716	85.3	3007560	83.3
Time since immigration				
Canadian-born	2407	76.5	8906000	71.1
10+ years	533	16.9	2591915	20.7
0-9 years	208	6.6	1019460	8.1
Education, highest level completed				
Below secondary school	93	2.9	769575	11.0
Secondary school diploma	395	12.4	1702160	24.3
Post-secondary school	2692	84.7	4547145	64.8
Employment status				
Employed	2513	79.2	16595030	60.9
Unemployed	152	4.8	1395050	5.1
Other	509	16.0	9269445	34.0
Total household income^b^				
Respondents	2539	100	4886655	100
<$20,000	116	4.6	556305	11.4
$20,000 -$39,000	214	8.4	831135	17.0
$40,000 -$59,000	310	12.2	824425	16.9
$60,000-$79,000	345	13.6	680850	13.9
$80,000-$99,000	393	15.5	552660	11.3
≥$100,000	1151	45.3	1441280	29.5
Number of children (under 18) in household				
1	1181	36.9	--	--
2-3	1874	58.5	--	--
≥4	150	4.7	--	--

^a^All Ontario data retrieved from: Statistics Canada, 2011 Census of Population

^b^Total household income, before taxes and deduction, from all sources in the past 12 months. Note that 21.1 % of the study sample did not provide their total household income

-- Comparable data for the Ontario population not available

### Physical activity

#### Guidelines

The proportion of parents that reported their child was reaching the Canadian Society for Exercise Physiology (CSEP) physical activity guidelines differed by the following child age groups: 1 to 4 years old (53.8 %, *n* = 253), 5 to 8 years old (72.9 %, *n* = 339), 9 to 12 years old (68.1 %, *n* = 312), and 13 to 17 years old (59.8 %, *n* = 400). Of these four age groups, only the 13- to 17-year-olds showed significant differences between the proportion of females (52.5 %) and males (67.5 %) meeting the guidelines (*p* = < .0001). Children under the age of one were excluded from the analysis, because there were no behavioural data collected for children in this group.

#### Parental support behaviours

The proportion of parents engaging in support behaviours for physical activity ranged from 80.5 % (*taking part in physical activity with child*), to 97.2 % (*encouraging child to be active outdoors with friends and family*) (Table 5).

**Table 5:** Proportion of parents engaging in support behaviour, by type

Type	Motivational	Instrumental	Regulatory	Conditional
Physical Activity (*n* = 2,237)	• 86.2 % encourage child to walk or cycle to places (e.g., friend’s houses, school) if they are reasonably close • 90.9 % encourage child to use resources in community to be active (e.g., parks) • 97.2 % encourage child to be active outdoors with friends and family	• 82.1 % enroll child in sports teams, clubs, or community-based programs where they can be active (e.g., Boys & Girls club, YMCA) • 93.7 % take child to places where they can be active		• 80.5 % take part in physical activities with child • 87.0 % watch child play sports or do other activities (e.g., martial arts, dance)
Healthy Eating (*n* = 2,288)	• 85.7 % encourage child to help choose and prepare snacks and meals • 98.5 % encourage child to eat breakfast	• 87.5 % serve raw vegetables and/or fruit for snacks between meals		• 34.3 % eat meals prepared or purchased at fast food restaurants with child • 83.8 % eat meals as a family away from the TV
Screen Time (*n* = 2,248)	• 86.2 % encourage child to limit their screen time during leisure time		• 78.5 % enforce rules about child’s screen time	• 79.9 % watch TV together as a family

#### Model

As indicated in Table 6, the following parental support behaviours contributed significantly to the predictions of child physical activity: parents who take their child to places where they can be active were more than twice as likely to have their child meeting physical activity guidelines (OR: 2.06; 95 % CI: 1.32-3.21); parents who encourage their child to be active outdoors with friends and family were 1.94 times more likely (95 % CI: 1.04-3.61); and parents who take part in physical activity with their child were 1.35 times more likely (95 % CI: 1.03-1.76). These associations remained significant in the model after adjusting for demographic and socioeconomic characteristics. Looking at the association of predicted probabilities and observed responses, the c-statistic, or area under the receiver operating characteristic (ROC) curve is 0.65, indicating a reasonably good model fit.

**Table 6:** Multivariable logistic regression predicting the likelihood that parents report their child reaches CSEP physical activity guidelines daily

Effect	Odds Ratio Point Estimate	95 % Wald Confidence Limits		*p-value*	
Intercept	--	--	--	0.8176	
Child age category (years)					
1 to 4	0.47	0.33	0.66	<.0001	***
5 to 8	1.32	0.97	1.80	0.0771	
9 to 12	1.19	0.90	1.58	0.2326	
13 to 17	1.00				
Child gender					
Male	1.28	1.05	1.57	0.0139	*
Female	1.00				
Parent age	0.98	0.97	1.00	0.0557	
Parent gender					
Male	1.00	0.79	1.26	0.9694	
Female	1.00				
Enroll child in sports teams, clubs, or community-based programs where he/she can be active (e.g., Boys & Girls club, YMCA)	1.32	0.98	1.77	0.0693	
Take child to places where he/she can be active	2.06	1.32	3.21	0.0014	*
Watch child play sports or do other activities (e.g., martial arts, dance)	0.94	0.67	1.32	0.7172	
Encourage child to use resources in community to be active (e.g., parks)	0.73	0.50	1.05	0.0851	
Take part in physical activities with child	1.35	1.03	1.76	0.0291	*
Encourage child to walk or cycle to places (e.g., friend’s houses, school) if they are reasonably close	1.12	0.84	1.51	0.4349	
Encourage child to be active outdoors with friends and family	1.94	1.04	3.61	0.0360	*

Association of Predicted Probabilities and Observed Responses

Area under the ROC curve (c) = 0.65

Significance code: *p < 0.05, **p < 0.01, ***p <0.001

### Healthy eating

#### Guidelines

The proportion parents that reported their child was reaching Canada’s Food Guide recommendations for FV consumption decreased as child age increased in the following age groups: 2 to 4 years old (50.3 %, *n* = 187), 5 to 8 years old (37.0 %, *n* = 183), 9 to 12 years old (16.2 %, *n* = 78), and 13 to 17 years old (8.2 %, *n* = 57). Within each age group, the proportion of children meeting FV guidelines did not differ significantly by child gender.

#### Parental support behaviours

The proportion of parents engaging in support behaviours for healthy eating ranged from 34.3 % (*eating meals prepared or purchased at fast-food restaurants with child*), to 98.5 % (*encouraging child to eat breakfast*) (Table 5).

#### Model

As shown in Table 7, the following parental support behaviours contributed significantly to the predictions of child FV consumption: parents who reported eating meals as a family away from the TV were 1.67 times more likely to report their child is meeting FV guidelines (95 % CI: 1.15-2.41); and parents who reported serving raw FV for snacks between meals were almost five times more likely (95 % CI: 2.67-9.12). These associations remained significant in the model after adjusting for demographic and socioeconomic characteristics. Looking at the association of predicted probabilities and observed responses, the c-statistic, or area under the ROC curve is 0.79, indicating a reasonably good model fit.

**Table 7:** Multivariable logistic regression predicting the likelihood that parents report their child reaches Canada's Food Guide recommendations for fruit & vegetables

Effect	Odds Ratio Point Estimate	95 % Wald Confidence Limits		*p-value*	
Intercept	--	--	--	<.0001	***
Child age category (years)					
2 to 4	12.56	8.11	19.44	<.0001	***
5 to 8	6.00	4.09	8.80	<.0001	***
9 to 12	2.03	1.37	3.02	0.0005	***
13 to 17	1.00				
Child gender					
Male	0.98	0.77	1.24	0.8687	
Female	1.00				
Parent age	1.00	0.98	1.02	0.9871	
Parent gender					
Male	0.66	0.49	0.87	0.0039	**
Female	1.00				
Eat meals prepared or purchased at fast food restaurants with child	0.78	0.61	1.01	0.0545	
Serve raw vegetables and/or fruit for snacks between meals	4.93	2.67	9.12	<.0001	***
Encourage child to help choose and prepare snacks and meals	1.39	0.95	2.02	0.0883	
Eat meals as a family away from the TV	1.67	1.15	2.41	0.0069	**
Encourage child to eat breakfast	1.22	0.33	4.44	0.7649	

Association of Predicted Probabilities and Observed Responses

Area under the ROC curve (c) = 0.79

*p < 0.05, **p < 0.01, ***p <0.001

### Screen time

#### Guidelines

The proportion of parents reporting that their child reaches the CSEP screen time guidelines differed by the following child age groups: 1 to 4 years old (15.3 %, *n* = 68), 5 to 8 years old (54.5 %, *n* = 249), 9 to 12 years old (37.7 %, *n* = 163), and 13 to 17 years old (29.7 %, *n* = 187). Significant gender differences were seen for the proportion of 9- to 12-year-old females (43.3 %) and males (32.3 %) meeting screen time guidelines (*p* = 0.018). The proportion of 13- to 17-year olds meeting these guidelines was also significantly different for females (39.4 %) and males (18.2 %) (*p* = < .0001).

As previously described, the total number of child screen time minutes were calculated by adding the time spent in front of a television and/or DVD player, computer or laptop, tablet or iPad®, and video game console (as reported by parents). To better understand the observed gender difference, screen time minutes for male and female children were looked at for each type of screen. As shown in Table 8, the largest gender difference is seen for average time spent playing video games in a day, for which males had approximately 28 min more than females.

**Table 8:** Summary of screen time minutes by child gender

Screen time, by type (minutes)	Females	Males	*Difference* (males - females)
Time spent watching TV/DVD	Mean = 77.18 SD = 59.68	Mean = 77.61 SD = 69.03	0.43
Time spent using the computer/laptop for non-homework related activities	Mean = 42.02 SD = 63.65	Mean = 50.00 SD = 82.49	7.98*
Time spent using tablet or Ipad	Mean = 34.26 SD = 56.71	Mean = 32.73 SD = 55.48	−1.53
Time spent playing video games	Mean = 6.59 SD = 27.36	Mean = 34.59 SD = 59.55	28.00*

* = *p* < 0.05

#### Parental support behaviours

Of the parents surveyed, 78.5 % reported enforcing rules about their child’s screen time, 79.9 % reported watching TV together as a family, and 86.2 % reported encouraging their child to limit their screen time during leisure time (Table 5).

#### Model

The following parental support behaviours contributed significantly to the predictions of child screen time behaviour: parents who enforce rules about their child’s screen time were 2.03 times more likely to have their child meeting screen time guidelines (95 % CI: 1.49-2.77); while families who watch TV together were 33.1 % less likely (95 % CI: 0.51-0.88) (Table 9). These associations remained significant in the model after adjusting for demographic characteristics.

**Table 9:** Multivariable logistic regression predicting the likelihood that parents report their child reaches CSEP screen time guidelines daily

Effect	Odds Ratio Point Estimate	95 % Wald Confidence Limits		*p-value*	
Intercept	--	--	--	0.0724	
Child age category (years)					
1 to 4	0.25	0.17	0.38	<.0001	***
5 to 8	2.22	1.62	3.06	<.0001	***
9 to 12	1.14	0.85	1.55	0.3848	
13 to 17	1.00				
Child gender					
Male	0.61	0.49	0.76	<.0001	***
Female	1.00				
Parent age	0.99	0.97	1.01	0.3681	
Parent gender					
Male	0.71	0.55	0.91	0.0075	**
Female	1.00				
Family watches TV together	0.67	0.51	0.88	0.0035	**
Encourage child to limit their screen time during leisure time	0.84	0.59	1.19	0.3179	
Enforce rules about child’s screen time	2.03	1.49	2.77	<.0001	***
Number of TVs in household	0.77	0.70	0.85	<.0001	***
Number of screens (other than TVs) in household	0.94	0.89	0.98	0.0080	**

Association of Predicted Probabilities and Observed Responses

Area under the ROC curve (c) = 0.75

*p < 0.05, **p < 0.01, ***p <0.001

The presence of every additional TV screen in the household decreased the likelihood of a child meeting screen time guidelines by 23.1 % (95 % CI: 0.70-0.85). Similarly, the presence of every additional screen (other than TV screens) in the household decreased the likelihood of a child meeting screen time guidelines by 6.3 % (95 % CI: 0.89-0.98). Looking at the association of predicted probabilities and observed responses, the c-statistic, or area under the ROC curve is 0.75, indicating a reasonably good model fit.

## Discussion

Overall, our results revealed that different types of parental support behaviours differentially predict whether children are meeting recommendations for physical activity, healthy eating, and screen time, as measured by parent report. All models were adjusted for the demographic and socioeconomic variables identified in Table 4; however, the focus of this discussion is on the role of parental support behaviours.

### Physical activity

Three parental support behaviours contributed significantly to predictions of child physical activity. First, parents who reported taking their child places where they can be active were more likely to have children meeting physical activity guidelines. This parental behaviour falls under the *instrumental* category of support, defined as the provision of aid and/or tangible services [16] (Table 2). Transportation, in addition to other instrumental support behaviours (e.g., provision of equipment), has previously been shown to be associated with increased physical activity in children [36]. Since opportunities for physical activity often involve venues away from one’s household, children may be dependent on parents for transportation to and from activities.

Parents who reported encouraging their child to be active outdoors with friends and family also emerged as a significant predictor of children meeting physical activity guidelines. Encouragement is a *motivational* support behaviour characterized by the provision of verbal or nonverbal prompts to engage in the behaviour, as well as validation of one’s involvement [16]. As proposed by Pugliese and Tinsley [35], parents’ motivational support may increase children’s competency and behavioural intentions, which, in turn, lead to increases in physical activity. The significance of this particular type of support over other motivational behaviours may be due to being active *outdoors*, with *friends and family*, or both.

Children who spend more time outdoors have been shown to be more active and have a lower prevalence of overweight than children spending less time outdoors [61]. In the same study, time spent outdoors positively predicted boys’ physical activity and inversely predicted the prevalence of overweight among older children 3 years later. Given the well-established decline in activity in adolescence, this finding highlights encouragement of outdoor activity as a promising support behaviour [61, 62].

The dynamic and rough landscapes for play that characterize natural environments are central to challenging children’s motor activity [63]. On top of these physical benefits, exercising in natural environments has been shown to be associated with greater positive engagement and decreased tension compared with exercising indoors [42]. Despite such strong support from the literature, children’s engagement in outdoor physical activity is impeded by a number of factors. A study by Clements [64] found that mothers identified their child’s TV viewing and computer game-playing as the top reason for a lack of outdoor play. Another barrier emphasized in the 2015 ParticipACTION Report Card on Physical Activity for Children and Youth is “the protection paradox” ([65], p. 7); whereby parents are increasingly overprotecting their children to keep them safe from external harms (e.g., crime, injury). We draw attention to the fact that 97 % of parents reported encouraging their child to be active outdoors with friends and family, which may be less indicative of the reality of children’s daily lives, and more reflective of parents’ recognition of the importance of outdoor play.

Third, our study found children were more likely to meet physical activity guidelines if parents reported taking part in physical activity with them. This involvement directly (or within proximity of the activity) with the child has been classified as *conditional* support [16]. The Youth Physical Activity Promotion Model proposed by Welk [66] acknowledges that engaging in active family activities may act to directly reinforce a child’s physical activity behaviour. This model suggests that parents also influence child behaviour indirectly through predisposing factors, including socialization and role modelling. It is possible that parent–child co-activity in early childhood strengthens child self-efficacy and fosters positive attitudes towards physical activity. These early predispositions, when enabled by a supportive environment and reinforced by social influences, collectively increase the likelihood that a child will be active on a regular basis [66]. With regard to interpreting the significance of shared physical activity, the field of neuroscience has presented strong evidence that social interaction has a powerful impact on neurogenesis. As Ratey and Hagerman [67] explain: “Exercise cues up the building blocks of learning, and social interaction cements them in place” (p. 262). Of all the parental support behaviours for physical activity explored in our study, participation with the child was reported least often (80.5 %). Our findings suggest that parent–child co-activity is a support behaviour warranting intervention aimed at increasing the proportion of children reaching physical activity guidelines. Future studies should consider evaluating changes in parent–child co-activity over time, and how this support behaviour relates to child physical activity.

### Healthy eating

Two parental support behaviours contributed significantly to predictions of child FV consumption in our model. First, parents who reported eating meals as a family away from the TV were more likely to have reported that their child was meeting FV guidelines. The implications of this conditional support behaviour can be interpreted through two lenses, by considering the evidence for “eating meals as a family” distinctly from evidence for “eating meals away from the TV.” Eating together as a family shapes the social context of meals, and children and adolescents who share family meals tend to eat more healthy foods and nutrients [38] and be in a normal weight range [41]. Key factors that appear to influence whether participation in family meals leads to a more healthful diet include: (1) healthy food availability at meals, (2) rules around meals, (3) health-related attitudes and concerns of family members, and (4) cultural factors, including the type of food cooked in the home [68].

Alternatively, eating meals in front of the TV can influence both *what* and *how much* children are consuming. Children from families in which television viewing is a normal part of meal routines may have diets that include fewer FV and more pizzas, snack foods, and sodas than the dietary patterns of children from families in which television viewing and eating are separate activities [42]. Screen-viewing may act as an environmental cue leading children to eat mindlessly—a behaviour which involves ignoring internal cues of satiety which signal when to stop eating [69]. A stimulus–response paradigm has also been used to demonstrate the influence of TV on diet; the longer a person is exposed to advertising, the more likely that person is to purchase and consume the advertised foods [42]. Regardless of whether this always holds true, eating family meals away from the TV appears to be an important factor that influences children’s dietary patterns.

Second, parents who reported serving raw FV for snacks between meals were almost five times more likely to have children meeting FV guidelines. This form of instrumental support contributes to the child’s eating environment by increasing FV availability and accessibility [26]. While these two concepts are highly connected, it is important to acknowledge that FV can be available (e.g., sitting in the fridge), but not accessible to children (e.g., not cleaned, peeled, chopped, etc.) [70]. Serving raw FV as a snack implies that the food is made available and accessible, and when it is easier to obtain FV, our results indicate that children are more likely to eat them. Other factors that should be considered in future studies include child food preferences, parental feeding styles, and the influence of the food environments outside of the home (e.g., schools).

### Screen time

In our logistic regression model, two parental support behaviours contributed significantly to predictions of child screen time behaviour. Parents who enforced rules about their child’s screen time were more likely to report that their child was meeting screen time guidelines. Enforcing rules and setting limits characterize the *regulatory* category of parental support. Our finding aligns with previous research demonstrating that setting rules to restrict child screen time has been associated with a lower probability of spending excessive time on screen-based activities [23]. To better understand the importance of rule-setting, future studies could consider: the types of rules parents are setting, how parents are introducing these rules, and if these rules are being consistently enforced.

Children were less likely to meet screen time guidelines if their parent reported that their family watches TV together. Co-viewing—a conditional support behaviour—may shape child behaviour through observational learning [47]. Watching TV as a family is a low-cost, home-based activity that can provide a reliable and positive setting for fostering family relationships [71]. Nonetheless, when the harms of sedentary behaviour are considered, habitual family co-viewing becomes problematic. Our finding is consistent with the screen time literature showing an association between parental co-viewing and increased TV time among young children and adolescents [23, 46, 49]. Family-based interventions should focus on replacing regular TV co-viewing with other shared family time activities which are more conducive to child health.

Finally, the number of TV screens in the household appeared to act as a barrier for meeting screen time guidelines, with every additional TV screen amplifying this relationship. Studies of the family environment—measuring the presence of a TV in the bedroom, or the number of additional TVs in the home—have also demonstrated an association between household TV access and TV viewing time [72, 73]. Similarly, the number of screens (other than TV screens) in the household was a negative predictor of children meeting screen time guidelines. The dose–response relationship consistently seen between screen access and screen time highlights the need for simple messages encouraging parents to limit the number of screens in the family environment.

### Limitations

Limitations of the current study include that all information was obtained from survey data, and therefore, measures of both parental support and child health were parent-reported. The former may contribute to self-report bias because of social desirability related to being a supportive parent. Self-report bias may also result from the latter, owing to the challenge of observing and accurately recalling all child health behaviours throughout the day. Accordingly, these parent-reported results may not necessarily represent actual rates in Ontario. For example, the results are much higher than the most recent Canadian estimates based on objective measures of child physical activity indicating that 9.3 % of children aged 5–17 are meeting guidelines [74]. Nevertheless, the aim of this study was not to establish prevalence, but rather to identify the types of parental support behaviours that were significantly correlated with these outcomes. Future research using objectively measured child health outcomes may better elucidate the nuances of the relationships reported here. This study also had a limitation relating to the parent-reported measure of child screen time. Although the survey contained a question on time spent using a cell phone in a typical day, there was concern about the validity of this survey item. As a result, cell phone time was not included the study, and thus, the amount of screen time reported is likely a conservative estimate. Another limitation of the analyses presented here is that the survey questions were not designed in accordance with the classification scheme for types of parental support behaviours used in this study (Table 2). Rather, the classification scheme was developed post hoc to organize the data. As such, when the parental support behaviours were classified by type of support (Table 3), a number of categories contained zero or few support behaviours. Future studies should consider including a range of items for each category of parental support to further test the relationships reported here.

## Conclusion

The results of the current study contribute to bridging a gap in knowledge about which types of parental support behaviours are most important for predicting child health behaviours. The framework of social support for youth physical activity developed by Beets et al. [16] provided a foundation of clearly defined parental support behaviours, from which this study’s classification scheme was adapted. Specifically, the *regulatory* category was added to account for behaviours that involve enforcing rules or setting limits. Given the inconsistent operationalizations of parental support behaviours in the literature, the adapted framework appears to be particularly useful for classifying different types of behaviours as they relate to child health behaviours including physical activity, healthy eating, and screen time. The importance of this adaptation is in the acknowledgment of the interdependence of different child health domains, and the identification of an instrument for future studies to theoretically frame social support, including parental support for child health as such. The classification scheme also allows for consideration of the effectiveness of different types of parental support behaviours between and within different child health domains. Its use is conducive to achieving greater consistency in the measurement, reporting, and understanding of how parents can best support their child’s health. However, at this time it would be premature to privilege any one parental support behaviour over others until a more complete analysis can be undertaken, as discussed above. What is clear from this research is that parental support behaviours are important determinants of child physical activity, healthy eating, and screen time. Thus, these support behaviours should be fully considered in any comprehensive approach to prevention and reduction of childhood overweight and obesity.

## Abbreviations

CATI, Computer Assisted Telephone Interview; CI, confidence interval; CSEP, Canadian Society of Exercise Physiology; FV, fruit and vegetable(s); MVPA, moderate-to-vigorous physical activity; ROC, receiver operating characteristic; WHO, World Health Organization

## Footnotes

1 The response rate was calculated from the number of people who participated, divided by the total number of eligible people in the total sample.

2 The cooperation rate was calculated from the number of people who participated, divided by the number of eligible people with whom contact was made.
